# Depressive Symptom and Related Factors: A Cross-Sectional Study of Korean Female Workers Working at Traditional Markets

**DOI:** 10.3390/ijerph14121465

**Published:** 2017-11-27

**Authors:** Won Ju Hwang, Jin Ah Kim, Sally H. Rankin

**Affiliations:** 1 College of Nursing Science, Kyung Hee University, 26 Kyungheedae-ro, Dongdaemun-gu, Seoul 02447, Korea; hwangwj@khu.ac.kr; 2 MacArthur Foundation Chair for Global Health Nursing, School of Nursing, University of California, N411Y, Box 0606, 2 Koret Way, San Francisco, CA 94143, USA; Sally.Rankin@ucsf.edu

**Keywords:** women workers, job stress, depression, marketplace

## Abstract

Background: Depression is one of the psychiatric diseases with a high prevalence rate, globally, and reportedly more prevalent among women than among men. Especially, women workers working at traditional markets are in depressive conditions without occupational health services. The purpose of this study is to investigate factors having a significant effect on the depressive symptoms of women workers at traditional markets in South Korea. Methods: A cross-sectional study was used and subjects for the present study were 500 female workers in three selected representative traditional marketplaces in South Korea. Results: The results of hierarchical regression analysis indicated that increased BMI (β = 0.297, *p* = 0.017), poor nutritional status (β = 0.596, *p* < 0.001), street vendor status (β = 2.589, *p* = 0.001), job stress (β = 0.491, *p* < 0.001), lower back pain (β = 0.377, *p* = 0.011), lower self-efficacy (β = −0.368, *p* = 0.002) and diminished family function (β = −0.633, *p* = 0.001) affected workers’ depressive symptoms. The explanatory power of these variables was 38.5%. Conclusions: Based on these results, future research should focus on incorporating theses significant factors into effective interventions designed to decrease depressive symptoms in this population. Moreover, this study will increase interest in occupational health nursing, particularly in relation to vulnerable social groups, and expand the scope of practice in the field.

## 1. Introduction

Depression is one of the psychiatric diseases with a high prevalence rate, globally, and is reported as not only degrading quality of life, but also resulting in high socioeconomic losses for communities and organizations [1]. Depression is prevalent in both the general population and among workers [2,3]. Since worker depression results in lower productivity, interest within social health sciences to prevent and manage worker depression is increasing. Furthermore, depression is reportedly more prevalent among women than among men [1]. The same can be applied to depression levels due to work stress, that is, female workers are also more vulnerable to depression because of a higher psychological burden, due to multiple social roles, and health problems due to these are reportedly increasing [4]. However, studies dealing with the mental health of female workers are limited. The few available studies on female workers’ mental health are concentrated on female shift workers [5,6] and female sex workers [7,8]. Moreover, previous studies on depression among female workers are limited to nurse practitioners [9,10], school nurses [11], and manufacturing workers [12] in Korea.

**Figure 1:**
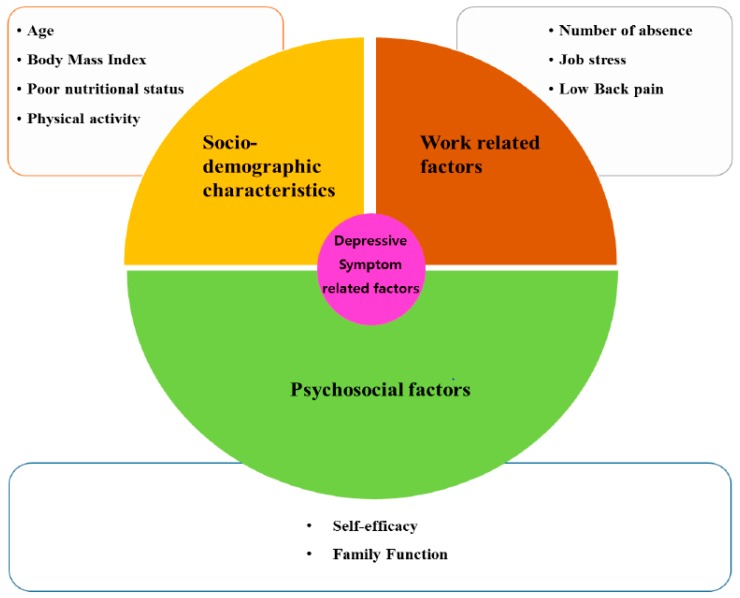
Conceptual framework of the study.

However, workers in traditional markets are mostly female. The female-worker ratio in three traditional markets in Seoul was 67.2%, higher than the economic activity participation rate of South Korean women, at 49.4% [13]. Moreover, individual characteristics such as gender [1,14], age [15], physical health and obesity [16] are associated with workers’ mental health. Consideration of psychological traits, including self-efficacy [17] and work-family conflicts [18], could be risk factors to workers’ mental health as well. Work-related factors experienced by workers can also contribute to workers’ mental health. Better mental health status is associated with lower levels of job stress [19], lower work demands [19] and less physical pain [20] (Figure 1). Furthermore, the World Health Organization (WHO) explained that an unfavorable physical work environment that features, for example, noise, dust, exposure to second-hand smoking, and heat increases the risk of depression [21]. This sub-group also does not receive systematic health management, despite being employed. Considering the work environments and mental health-related factors of women working in traditional markets, where there is higher percentage of street vendors in low socioeconomic status than merchants in stores, there is seemingly a need for research on depression levels and the related factors and intervention studies.

Accordingly, this study aimed to determine depressive symptom levels among female workers at traditional markets, to identify an effective intervention approach, by determining factors affecting depressive symptoms and to subsequently provide baseline data for future health promotion development.

## 2. Methods

### 2.1. Data Collection

The present study was a descriptive survey, aimed at determining demographic characteristics, health-related characteristics and work-related characteristics according to depressive symptom levels among female workers in traditional markets. Data were collected after the Institutional Review Board at K University granted permission (KHSIRB-13-035). Subjects for the present study were 503 female workers in three selected representative traditional markets in Seoul, South Korea, selling fruit and vegetables, agricultural products, fish, stock fish, and medicinal herbs. Five hundred responses, excluding three that could not be processed due to too many unanswered questions, were analyzed in this study. Thus, response rate of the study was 99.4%. To determine the number of stores and tradespeople within each market, interviews were conducted with presidents/representatives of merchant groups or directors of all markets’ management offices. The subjects were adult women over 20 years old and unmarried people were excluded to confirm the family function. The interview time was between 1 and 3 pm on weekdays in the markets’ management office to minimize the interference of the customers and noise. Also, the street vendors defined the business as a shop selling on four sides where there is no wall, and the others as a store merchant. Three trained researchers visited stores and street stalls at each market, to conduct individual interviews for 500 female workers from October 2014 to January 2015. Owing to the study’s purpose, confidentiality and the fact that the data would be used only for the stated purposes, signed agreements were collected from female tradespeople who understood the study’s purpose and volunteered participation. Data were collected through a structured survey administered through interviews, each taking about 15–20 min.

### 2.2. Questionnaire Investigation

#### 2.2.1. Depressive Symptom

Depressive symptoms were measured using the Korean version of the Center for Epidemiologic Studies Depression (CES-D) Scale [22,23]. In the West, the criteria for probable and definite depression are generally 16 and 25 points, respectively. In a depression epidemiological survey on South Koreans by Cho and Kim in 1993 [23], investigators suggested using 21 points as the baseline cut-off for screening for suspected depression and 25 points for screening for definite depression. Accordingly, in the present study, definite depression was classified as ≥25 points, suspected depression as ≥21, and probable depression as ≥16. Finally, the current study adopted the cut-off point of 21 to define depressive symptoms. The CES-D comprised 20 questions. A higher score represents higher depressive symptom levels. The Cronbach’s alpha of CES-D’s in the previous study was 0.90 [8] and 0.86 in the current study.

#### 2.2.2. Physical Activity

A short version of the International Physical Activity Questionnaire (IPAQ) [24] was used. The data were classified into three groups (inactive, minimally active, and HEPA/health enhancing physical activity active), according to the IPAQ scoring protocol. This study’s Cronbach’s alpha was 0.68.

#### 2.2.3. Over-Commitment

Over-commitment, a component of job stress, was measured through the Korean version of the over-commitment component of the Effort-Reward Imbalance (ERI) [25]. Over-commitment in the ERI instrument comprises six questions. Higher scores indicate higher over-commitment. The Cronbach’s alpha was 0.71 in the present study.

#### 2.2.4. Emotional Labor

To measure the level of emotional labor, an instrument developed by Morris and Feldman’s research [26] was used. It comprised nine questions that were sub-factors of emotional labor; three questions were on the frequency of emotional expression, three on the carefulness of emotional expression, and three on emotional dissonance. A higher score meant greater emotional labor. Cronbach’s alpha was 0.76 in the current study.

#### 2.2.5. Self-Efficacy

Self-efficacy was measured through the Korean Adaptation of the General Self-Efficacy Scale [27]. The instrument comprises ten questions. The total score range was 10–40 and a higher score means higher self-efficacy. Cronbach’s alpha was 0.75 at development [27], and 0.77 in the current study.

#### 2.2.6. Family Function

Family function was measured using the Family APGAR Questionnaire [28]. The instrument comprised five questions on adaptation, partnership, growth, affection, and was resolved on a three-point scale. The total score of 7–10 represents high family function, 4–6 represents moderate family dysfunction, and 0–3 represents extreme family dysfunction. Reliability, as shown by Cronbach’s alpha, was 0.72 at development and 0.80 in the present study.

#### 2.2.7. Nutritional Status

To evaluate nutrition status, the US-developed Nutrition Screening Initiative (NSI) checklist [29] was used. The NSI comprises ten questions. A score of 0–2 indicated a good nutrition, 3–5 indicated a moderate nutritional risk, and ≥6 indicated a high nutritional risk. Reliability in this study measured through Cronbach’s alpha was 0.63.

#### 2.2.8. Lower Back Pain

To measure lower back pain, the Visual Analogue Scale (VAS) [30] was used, stating, “Please mark the degree of lower back pain that you feel now on the line below”. The scale’s left and right ends represent no pain (0) and the most severe pain (10), respectively; a higher score means stronger pain.

### 2.3. Data Analysis

Data were analyzed using SPSS 22.0 (SPSS Inc., an IBM Company, Seoul, Korea). Frequency and percentages were obtained on depressive symptom levels, demographic characteristics by depressive symptom level, and health- and job-related characteristics of the women working in traditional markets. Pearson’s correlation was conducted on the relationship between depressive symptoms and general characteristics and health- and job-related characteristics. Hierarchical regression analysis was performed to determine the associations of age, Body Mass Index (BMI), nutritional status, physical activity, store type, number of absences, job stress, back pain, self-efficacy, and family function on depressive symptoms. Multicollinearity, residuals, and singular values were examined to enable use of hierarchical regression analysis. To verify independence disturbances, the Dubin-Watson statistics on the final model was 1.393, showing no problems with autocorrelation. Moreover, there was no multicollinearity, with the test revealing tolerance of 0.772–0.960, ranging between 0.1 and 1.0 the variance inflation factor was 1.042–1.295, not exceeding the criterion of 10.0. Linearity, normality, and homoscedasticity assumptions were satisfied. Further, the maximum value of Cook’s distance, obtained to check for singular value, was 0.068, showing no singular value, since none exceeded 1.0. Therefore, the study’s final regression equation satisfied all the assumptions for the regression equations, and the regression analysis results were reliable.

## 3. Results

### 3.1. Socio-Demographic Characteristics and Work-Related Factors by Depressive Symptom Level

The average depressive symptom level, measured through the CES-D, was 17.3 (±8.1; range: 0–60). When the respondents were divided into depressive symptom and non-depressive symptom groups, using a CES-D score of ≥16, a widely used cut-off point in the West, 52.9% were classified depressed. In the previous study [24], however, since the investigators suggested 21 points as the baseline cut-off for screening suspected depression and 25 points for screening definite depression among South Koreans, the current study adopted the cut-off point of 21. Thirty-one percent were classified depressed under the suspected depressive symptom group; when the cut-off point was 25 points, 21.0% were classified under the definite depressive symptom group. The participants’ average age was 59.2 years. In the depressive symptom group, 32.9% were ≥65 years, more than those aged <65 years, at 30.2%; this difference was not statistically significant. There were more overweight (27.1%) and obese (43.4%) subjects in the depressive symptom group than were subjects with a normal body mass index (BMI) (25.1%) (*p* = 0.002). Regarding subjective health perception, more subjects who considered themselves unhealthy (40.2%), as opposed to those who did not (25.6%) (*p* = 0.037), were in the depressive symptom group. Moreover, family function (*p* < 0.001), nutritional status (*p* < 0.001), and self-efficacy (*p* < 0.001) were low (or poor) in the depressive symptom group, compared to the non-depressive symptom group. The depressive symptom group had more subjects with ≥8 total working hours (32.4%) than those with <8 h (21.0%) (*p* < 0.001); street vendors (55.4%) had more depressive symptom than those in stores (21.3%). Furthermore, the lower back pain score was higher for the depressive symptom group, compared to the non-depressive symptom group (*p* < 0.001). Subjects with longer careers had lower depressive symptom levels, but these were not statistically significant (Table 1).

### 3.2. Correlation between Independent Variables and Depressive Symptom

Depressive symptoms positively correlated with BMI (*r* = 0.137, *p* = 0.002), nutritional status (*r* = 0.385, *p* < 0.001), job stress (*r* = 0.089, *p* = 0.047), and lower back pain (*r* = 0.257, *p* < 0.001); but negatively correlated with self-efficacy (*r* = −0.295, *p* < 0.001) and family function (*r* = −0.384, *p* < 0.001) (Table 2).

### 3.3. Factors Associated with Depressive Symptom

A three-step hierarchical multiple linear regression analysis was performed to test factors associated with depressive symptom among study participants. Step 1’s analysis included socio-demographic factors such as age, BMI, nutritional status, and physical activity; step 2’s analysis included work-related factors such as store type, absent days per year, job stress, and lower back pain; and step 3’s analysis included psychosocial factors such as self-efficacy and family function. Step 1’s analysis showed that BMI (β = 0.351, *p* = 0.007) and nutrition (β = 0.824, *p* < 0.001) were associated with depressive symptoms and explained 22.0% of depressive symptom (*R*^2^ = 0.220, *p* < 0.001).

Step 2’s analysis showed that BMI (β = 0.269, *p* = 0.036), nutrition (β = 0.692, *p* < 0.001), street vendor status (β = 2.784, *p* < 0.001), job stress (β = 0.466, *p* < 0.001), and lower back pain (β = 0.545, *p* < 0.001) were significantly associated with depressive symptom, and explained 32.5% of depressive symptom, which was higher than that in step 1.

Lastly, step 3’s analysis showed that 38.5% of depressive symptom was explainable and BMI (β = 0.297, *p* = 0.017), nutrition (β = 0.596, *p* < 0.001), street vendor status (β = 2.589, *p* = 0.001), job stress (β = 0.491, *p* < 0.001), lower back pain (β = 0.377, *p* = 0.011), self-efficacy (β = −0.368, *p* = 0.002), and family function (β = −0.633, *p* = 0.001) were associated with depressive symptoms (Table 3). Therefore, work-related factors and psychosocial factors were associated with depressive symptoms more than personal factors were.

## 4. Discussion

As workers’ depression reportedly influences workers’ health, productivity, and socio-economic losses, interest in the prevention and management of depression about the workers is increasing. Depression levels due to job stress are higher among female workers than among males, and female workers are more exposed to depression because of a higher psychological burden resulting from various social roles; health problems caused by these are reportedly increasing [4]. In particular, because female workers in traditional markets are prone to depressive symptoms, given the high female-worker ratio and the poor working environments, such as street stalls, efforts aimed at resolving these issues are necessary. Therefore, this study aimed to determine depressive symptom levels and factors associated with depressive symptoms among female workers in traditional markets.

First, the mean depressive symptom level (CES-D) was 17.3, higher than the 13.8 in the study on female workers in the manufacturing industry [31]. Moreover, when compared to the 36.3% probable depression prevalence among nurse practitioners [9], the probable depressive symptom prevalence in the current study was 1.5 times higher than the previous study of 52.9%. Therefore, it is evident that depression management and intervention programs are needed for this vulnerable population.

Second, BMI and poor nutritional status among socio-demographic factors were statistically associated with depressive symptoms in this study. Many studies report that obesity influences depression; a systematic review [32] also showed that the depression risk among obese women is almost double that than among normal weight people. Previous study that investigated the relationship between depression and weight, using the national health and nutrition survey data of 2005–2006 in the US, showed a four times higher depression prevalence in obese women [33]. The current study also showed that high BMI was associated with depressive symptoms, which is similar to previous studies [32,33]. Furthermore, the current study found no association of physical activity on depressive symptoms. There are studies arguing that physical activity influences depression while others find that physical activity has no influence [34]. This discrepancy could be because such studies did not clearly identify the physical activity domain as including labor, hobbies, and household chores; indeed, McKercher et al. [34] stated that the relationship between workplace activities and depression must be clarified. Since the short IPAQ version [24] used in the present study does not clearly distinguish between physical activity sub-levels, definite conclusions about physical activity’s influence on depression are not possible. Therefore, future study should investigate physical activity and depression in-depth, using the full version of IPAQ with itemized physical activity sub-levels. The study sample’s average score for nutrition was 7.87, indicating severe nutrition risks. Since the average age of female workers in traditional markets is 60 years, the risks of lack of appetite, tooth loss, reduced bodily utilization of nutrients, illness onset and recovery, and the potential effects of age on mental health, including depression, should be considered [35]. Furthermore, the nutritional status of this study population is found to be poor, given a Korean study, which found that 34.3% of 177 petty merchants at traditional markets consumed ≥1 meal of fast food daily. Therefore, given findings that poor nutritive conditions affect depression [36], the current participants’ irregular eating habits or poor nutritive conditions, lacking set mealtimes and often eating packaged instant or delivered food, seem closely related to depression. Accordingly, there is a need for further analysis of factors affecting this study population’s nutrition and implementation of related interventions. According to the previous study on the relationship between socioeconomic status and the risk of major depression [37], low household income had a higher incidence of major depression than others. In this study, the question about household income was included in the questionnaire, but it was not available for analysis because of high missing data rate (47.3%). This is because many of the factors related to personal income such as season, weather, and national events, are not fixed in one month’s income. Also, participants were embarrassed to reveal their low income. Therefore, further study should be conducted to elucidate the effect of income on depressive symptom in workers at traditional market place.

Third, the work-related factors, such as store type, job stress, and lower back pain, were significantly associated with depressive symptoms among traditional market workers. This study showed that the percentage of street vendors was almost 35%. This means that workers at traditional market places are more easily exposed to a poor physical environment. The current findings matched those of a healthy workplace framework of Burton and WHO [22] showing that a poor work environment featuring dust, noise, heat, and cold, based on seasonal changes, has workers who are more prone to mental health issues. Moreover, an imbalance between effort and reward and over-commitment as work-related stress, ultimately influencing mental health as job stress [25,38]. The results of the current study correspond with those of previous study. The job stress results in the current study are higher than that in a Japanese study that sampled office workers [39]. Considering the work characteristics of female workers in traditional markets, which require them to do all aspects of the work by themselves, they may show more job stress than do general office workers. This study found that the musculoskeletal symptoms such as lower back pain were associated with depressive symptoms, similar to Beesdo et al.’s findings on depression and pain [40], demonstrating that pain is closely related to depression because it reduces quality of life and job efficiency, and also increases the access of medical services. Furthermore, the previous study showed significantly strong correlations between chronic lower back pain and depression [20] supporting the current results. In particular, lower back pain is a common work-related health problem, with repeated bending or a twisting posture as the primary reason. Therefore, female workers in traditional markets are prone to chronic lower back pain due to repeated job activities in a tight space and an incorrect work posture. Since this might increase the risk of depressive symptoms, a countermeasure for this problem is necessary.

Fourth, self-efficacy and family function among the psychosocial factors were associated with depressive symptoms in the participants of this study. Lower self-efficacy and its contribution to depression and anxiety has long been supported theoretically [41]. Similarly, social support’s (including family support) reduction of depression has been proven theoretically [42]. The family function score of female workers in traditional markets indicates extreme family dysfunction, the workers’ resultant predisposition towards depression poses a serious concern. In particular, the results show difficulties in simultaneously assuming occupational and household roles. The resultant work-family conflict, with women experiencing higher psychological conflict than men do [43], coupled with findings that work-family conflict correlates with mental health issues [44]. Therefore, it is necessary to determine if family function decreases due to work-family conflict among female workers at traditional markets with a relatively long average career spanning 19 years. Further research on comprehensive reasons for family dysfunction within this study population is needed.

In summary, this study found that depression among female workers at traditional markets is closely related to socio-demographic characteristics such as age, BMI, and nutritional status, work-related factors such as the work environment, job stress, and lower back pain, and psychosocial factors such as self-efficacy and social support, including family support. However, according to the previous study [45], 89.2% of women workers in traditional markets are not insured. Moreover, in the occupation safety and health acts of Korea, health managers must be mandatory in the case of workplaces with 50 or more employees, but this sub-group has not received systematic health management because of the absence of health managers and social consideration, despite being employed. Based on the results, we are developing depression intervention programs for female workers at traditional markets, including psychological health care such as counseling and meditation, and musculoskeletal health care such as yoga, stretching, line dance, and education for working posture. This depression intervention is also including family support and social network group building program. However, the effects of these multicomponent programs need to be verified, and future research is needed to support and expand these findings.

## 5. Conclusions

The study findings enable the development of interventions and social support means for preventing depressive symptoms among female workers in traditional markets, by determining depressive symptom levels within this group, which have not been studied before, and by comprehensively analyzing the influencing factors in terms of depression, including socio-demographic characteristics, work-related and psychosocial factors. Furthermore, this study will increase interest in occupational health services, particularly in relation to vulnerable social groups, and expand the scope of practice in the field.

The study’s limitations are as follows. First, a convenience sampling method was used and subject selection was limited to one city, rendering generalization difficult. Second, this was a cross-sectional study, meaning that cause-and-effect relationships between the variables cannot be inferred. Moreover, even though this study used both continuous and categorical factors as the independent variables, all variables were listed in parallel and showing their relationships with depressive symptom. There was no multicollinearity, with the test revealing tolerance of 0.772–0.960, ranging between 0.1 and 1.0. The variance inflation factor was 1.042–1.295, not exceeding the criterion of 10.0. However, these factors could influence each other. Thus, further research using more advanced statistical analysis is needed to confirm the related factors with women workers’ depression symptoms in traditional markets. Third, even though the instruments used were standardized, data collection through individual interviews may mean that the surveyor’s attitude could have influenced respondents’ subjective thoughts; thus, response biases cannot be excluded. Fourth, since the short IPAQ version used in this study does not clearly distinguish between physical activity sub-levels, no clear conclusions about physical activity’s influence on depressive symptoms can be made. Therefore, in-depth investigation on physical activity and depressive symptoms, through the full version of IPAQ comprising itemized physical activity sub-levels, is necessary. Furthermore, although income is a major factor affecting depression, it has not been used in this study, because of the high missing data rate. Thus, further study should be conducted to elucidate the effect of income on depressive symptoms in workers at traditional markets. Finally, the study took place from October to January, when seasonal variations such as cold weather could have impacted depression symptom. Thus, future research comparing cold weather and hot weather seasons is necessary to substantiate and expand the findings of the present study.

## Figures and Tables

**Table 1:** Distribution of depressive symptom group according to socio-demographic and work related factors (*N* = 500).

Variables	Number (%)/Mean ± SD	Depressive Symptom Level		*p*-Value
		Normal Group (*n* = 345) *N* (%)/Mean ± SD	Depressive Symptom Group (*n* = 155) *N* (%)/Mean ± SD	
**Age (year)**				
<65	351 (70.2)	245 (69.8)	106 (30.2)	0.311
≥65	149 (29.8)	100 (67.1)	49 (32.9)	
**Body Mass Index**				
Underweight	3 (0.6)	3 (100.0)	0 (0.00)	0.002
Normal	211 (43.0)	158 (74.9)	53 (25.1)	
Overweight	155 (31.6)	113 (72.9)	42 (27.1)	
Obese	122 (24.8)	69 (56.6)	53 (43.4)	
**Subjective health status**				
Healthy	215 (43.0)	160 (74.4)	55 (25.6)	0.037
Fair	203 (40.6)	136 (67.0)	67 (33.0)	
Unhealthy	82 (16.4)	49 (59.8)	33 (40.2)	
**Physical activity**				
Inactivity	87 (17.4)	60 (69.0)	27 (31.0)	0.252
Minimally activity	89 (17.8)	55 (61.8)	34 (38.2)	
Health enhancing physical-activity	324 (64.8)	230 (71.0)	94 (29.0)	
**Store type**				
Shop	296 (68.0)	233 (78.7)	63 (21.3)	<0.001
Street vendor	139 (32.0)	62 (44.6)	77 (55.4)	
**Job tenure (year)**				
<10	155 (31.0)	104 (67.1)	51 (32.9)	0.592
10–20	151 (30.2)	102 (67.5)	49 (32.5)	
20≤	194 (38.8)	139 (71.6)	55 (28.4)	
**Working time (hour/day)**				
<8	62 (12.4)	49 (79.0)	13 (21.0)	0.044
8≤	438 (87.6)	296 67.6)	142 (32.4)	
Age	59.23 ± 9.90	58.88 ± 9.80	60.01 ± 10.11	0.238
CES-D	17.33 ± 8.14	12.81 ± 4.41	27.38 ± 4.91	<0.001
Probable depression (CES-D ≥ 16)	500 (100)	236 (47.2)	264 (52.8)	
Suspicious depression (CES-D ≥ 21)	500 (100)	345 (69.0)	155 (31.0)	
Definite depression (CES-D ≥ 25)	500 (100)	395 (79.0)	105 (21.0)	
Family Function	3.69 ± 2.21	4.14 ± 2.18	2.68 ± 1.91	<0.001
Nutritive conditions	7.87 ± 1.50	7.41 ± 1.00	8.87 ± 1.89	<0.001
Self-efficacy	27.01 ± 3.33	27.73 ± 2.99	25.42 ± 3.50	<0.001
Job stress (Over-commitment)	17.90 ± 3.52	17.79 ± 3.54	18.15 ± 3.49	0.275
Lower back pain	3.54 ± 2.44	3.19 ± 2.45	4.30 ± 2.24	<0.001
Number of Absence	3.94 ± 45.43	4.48 ± 54.08	2.75 ± 12.27	0.075

**Table 2:** Correlations among independent variables and depressive symptom (*N* = 500).

Variables	1	2	3	4	5	6	7	8	9	10
1. Depressive symptom	-									
2. Age	0.064	-								
3. Body Mass Index	0.137 **	0.143 **	-							
4. Poor nutritional status	0.385 **	0.152 **	0.108 *	-						
5. Physical Activity	−0.085	−0.032	−0.082	−0.231 **	-					
6. Number of Absence	0.084	0.018	0.053	0.046	−0.033	-				
7. Job Stress	0.089 *	0.078	−0.049	−0.026	0.228 **	0.068	-			
8. Low Back Pain	0.257 **	0.227 **	0.115 *	0.213 **	0.075	0.021	0.124 **	-		
9. Self- efficacy	−0.295 **	−0.089 *	−0.009	−0.190 **	0.064	0.014	0.071	−0.181 **	-	
10. Family Function	−0.384 **	−0.129 **	−0.114 *	−0.252 **	−0.072	0.037	0.080	−0.224 **	0.368 **	-

* *p* < 0.05, 2 tailed, ** *p <* 0.01, 2 tailed.

**Table 3:** Multiple regression analyses of selected variables on depressive symptom (*N* = 500).

Variables	Model I			Model II			Model III		
	β	t	*p*	β	t	*p*	β	t	*p*
Age (year)	0.043	1.146	0.252	0.003	0.088	0.930	−0.025	−0.658	0.511
BMI	0.351	2.725	0.007	0.269	2.100	0.036	0.297	2.397	0.017
Poor nutritional status	0.824	9.409	<0.001	0.692	7.598	<0.001	0.596	6.706	<0.001
Physical activity	0.000	0.643	0.520	−0.000	−1.077	0.282	0.000	−1.368	0.172
Store type (Shop/Street vendor)				2.784	3.537	<0.001	2.589	3.425	0.001
Number of absences (days/year)				0.058	1.543	0.124	0.060	1.671	0.096
Job stress				0.466	4.340	<0.001	0.491	4.769	<0.001
Low back pain				0.545	3.600	<0.001	0.377	2.546	0.011
Self-efficacy							−0.368	−3.152	0.002
Family function							−0.633	−3.461	0.001
Intercept	1.841			−6.678			7.517		
F	28.217			20.891			21.633		
*R*^2^	0.220			0.325			0.385		
Adjusted *R*^2^	0.212			0.310			0.368		
*R*^2^ change	0.220			0.105			0.060		
*p*	<0.001			<0.001			<0.001		
